# The repurposing of type I-E CRISPR-Cascade for gene activation in plants

**DOI:** 10.1038/s42003-019-0637-6

**Published:** 2019-10-18

**Authors:** Joshua K. Young, Stephen L. Gasior, Spencer Jones, Lijuan Wang, Pedro Navarro, Becca Vickroy, Rodolphe Barrangou

**Affiliations:** 1 Department of Molecular Engineering, Corteva Agriscience™, Johnston, IA 50131 USA; 2 Department of Food, Bioprocessing and Nutrition Sciences, North Carolina State University, Raleigh, NC 27695 USA

**Keywords:** Gene regulation, Plant genetics, Genetic engineering

## Abstract

CRISPR-Cas systems are robust and facile tools for manipulating the genome, epigenome and transcriptome of eukaryotic organisms. Most groups use class 2 effectors, such as Cas9 and Cas12a, however, other CRISPR-Cas systems may provide unique opportunities for genome engineering. Indeed, the multi-subunit composition of class 1 systems offers to expand the number of domains and functionalities that may be recruited to a genomic target. Here we report DNA targeting in *Zea mays* using a class 1 type I-E CRISPR-Cas system from *S. thermophilus*. First, we engineer its Cascade complex to modulate gene expression by tethering a plant transcriptional activation domain to 3 different subunits. Next, using an immunofluorescent assay, we confirm Cascade cellular complex formation and observe enhanced gene activation when multiple subunits tagged with the transcriptional activator are combined. Finally, we examine Cascade mediated gene activation at chromosomal DNA targets by reprogramming *Zea mays* cells to change color.

## Abstract (web summary)

Class 2 CRISPR-Cas systems are widely used in genome editing. Joshua Young1, Stephen Gasior et al. now engineer the type I-E Cascade complex from S. thermophiles, optimize the system for expression and nuclear localization in Zea mays and show it compares favorably with Cas9 as a RNA-guided transcriptional activator.

## Introduction

CRISPR (clustered regularly interspaced short palindromic repeat)-Cas (CRISPR associated) systems have emerged as potent genome editing tools^1^. Guided by a small RNA that base pairs with a DNA target in the vicinity of a short protospacer adjacent motif (PAM), these enzymes offer unprecedented robustness and re-programmability^2,3^. As a tool for genome editing, they have been used to efficiently introduce targeted DNA double-strand breaks (DSBs) in eukaryotic chromosomal DNA^4,5^. Then, by harnessing endogenous DNA repair pathways, favorable modifications (e.g., those that enhance plant yield^6^ or repair disease-causing alleles^7^) can be engineered. New applications utilizing nuclease-deficient or impaired Cas proteins as RNA-guided DNA-binding platforms have emerged. For these, additional accessory protein domains are tethered to the amino and carboxyl termini or guide RNA (gRNA) bringing novel functionalities to the Cas-gRNA complex. Such fusions have been used to upregulate or downregulate gene expression^8^, deaminate DNA^9^, modify the epigenome^10^, and visualize genomic loci^11^.

Until very recently^12^, only class 2 CRISPR-Cas systems, comprised of a single effector protein (type II-Cas9 and type V-Cas12)^13^, have been adopted as eukaryotic genome editing tools. In contrast, the most abundant and diverse CRISPR-Cas systems belong to class 1^14^. These systems, defined as type I and III, encode multi-subunit complex effectors^13,14^. In the case of type Is, a CRISPR-associated complex for antiviral defense (Cascade)^15^, comprised of 3–5 proteins (depending on the subtype), facilitates RNA-guided DNA target recognition^16^. Once in complex with the target, a processive single stranded exonuclease, Cas3, is recruited to and activated by the Cascade complex to perform target DNA destruction^17^. Similar to Cas9 and Cas12 proteins, type I Cascade recognizes a PAM sequence providing self vs. non-self distinction in its native setting^18–20^. In type I-E systems, PAM recognition by Cascade is accomplished by the CasA protein^21^ and is typically comprised of 1–3 nucleotides located upstream of the protospacer^19,20,22^. The guide RNA (gRNA) of Cascade is a single CRISPR RNA (crRNA) which consists of a spacer flanked on either side by a portion of the CRISPR repeat^15,16,22^.

For genome editing applications, Cascade provides unique advantages over single effectors. First, a component of Cascade, CasE, is a ribonuclease that provides inherent guide RNA processing^15^. This enables the co-transcription of several guide RNAs from a single promoter, as well as the use of promoters without a defined transcriptional start site such as many polymerase II promoters. Although some Cas12 effectors have evolved this capability, Cas9 has not^23^. Second, Cascade crRNA target recognition typically spans ~30 bp or greater^24,25^ offering the potential for high specificity in larger and more complex eukaryotic genomes. Next, the processive single stranded DNA exonuclease associated with type I systems, Cas3, provides a new approach for the introduction of large chromosomal deletions adjacent to Cascade DNA targets^13^. Finally, being comprised of 8–11 proteins, Cascade provides multiple structural options for the attachment and recruitment of accessory domains with new functionalities.

To our knowledge, we report the first use of type I CRISPR-Cascade to modulate gene expression in eukaryotic cells. First, using a rapid trans-activation immunofluorescent reporter, we establish robust gene activation configurations for the type I-E Cascade complex from *Streptococcus thermophilus*. Next, we demonstrate it can be used as a robust RNA-guided DNA binding platform which performs competitively in comparison to the Cas9 from *Streptococcus pyogenes*, highlighting potential advantages over class 2 effectors. Finally, we confirm complex formation in the nucleus by activating a chromosomal gene that results in anthocyanin biogenesis perturbing the color phenotype of *Zea mays* aleurone cells.

## Results

### Engineering type I SthCascade for gene activation

**Fig. 1:**
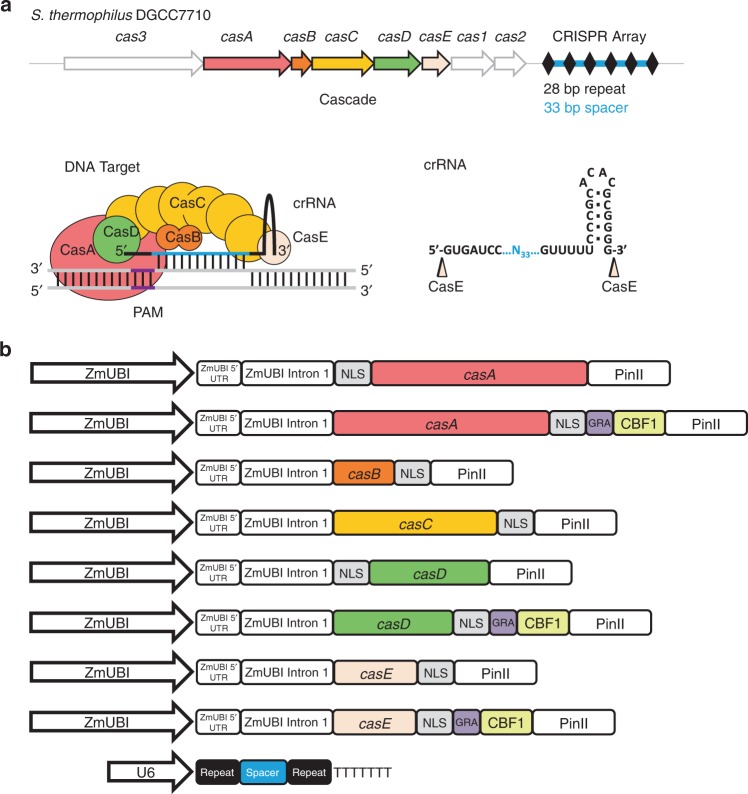
Engineering type I-E Cascade complex formation in *Zea mays*. **a** Native type I-E CRISPR-Cas locus from *S. thermophilus* DGCC7710. CRISPR repeats are indicated with a black diamond and the spacer region is in blue. CasE cleaves the primary CRISPR RNA (crRNA) transcript after the hairpin in the CRISPR repeat yielding the mature crRNA depicted. The Cascade complex guided by the crRNA in the vicinity of a 5′ PAM recognizes a double stranded DNA target as illustrated. **b** *Zea mays* optimized Cascade and crRNA expression constructs resulting in Cascade complex formation and gene activation. Zm *Zea mays,* UBI ubiquitin, NLS nuclear localization signal, PinII potato proteinase inhibitor terminator, CBF1 sequence encoding C-terminal acidic transcriptional activation domain from *Arabidopsis* cold binding factor 1

The type I-E Cascade genes, *casA*, *casB*, *casC*, *casD*, and *casE*, from *Streptococcus thermophilus* DGCC7710 (Sth) responsible for CRISPR RNA (crRNA) maturation (CasE) and RNA guided DNA target recognition (CasA, CasB, CasC, CasD) (Fig. 1a)^22^, termed Cascade, were engineered for expression, nuclear localization, and transcriptional gene activation in *Zea mays* (Zm). First, genes were optimized, adjusted for ideal GC content, and repetitive sequences and gene destabilizing features such as miniature inverted-repeat transposable element (MITE) sites removed where possible. Next, a sequence encoding a nuclear localization signal (NLS) from Simian Virus 40 (SV40) was attached to each of the genes. To minimize impact on Cascade complex activity and ensure NLS recognition, the structure of the type I-E Cascade complex from *E. coli* (Eco) was referenced^26^ and associated termini likely to be not buried in the SthCascade complex were selected as NLS attachment points. Next, a sequence encoding the C-terminal acidic plant transcriptional activation domain from the *Arabidopsis* cold binding factor 1 (CBF1)^27^ was codon optimized and separately fused in-frame to the 3′ end of the *casA*, *casD*, and *casE* genes, using a sequence encoding a short protein linker, GRA^28^ (Fig. 1b). CasA, CasD, and CasE were selected for CBF1 attachment as they represent monomeric subunits in the SthCascade complex and simplify experiments aimed at comparing gene activation potential with Cas9. Next, to facilitate robust and constitutive protein expression, the resulting genes were cloned into expression cassettes consisting of a Zm ubiquitin (UBI) promoter, intron, and 5′ untranslated region (UTR) and potato proteinase inhibitor (PinII) terminator as described previously for *Streptococcus pyogenes* (Sp) Cas9^29^ (Fig. 1b).

Next, the guide RNA for SthCascade was engineered for expression and targeting in *Zea mays*. In the *S. thermophilus* type I-E CRISPR-Cas system, the guide RNA is comprised of a single CRISPR RNA (crRNA). It contains a 33 nt length spacer flanked on either side by a portion of the CRISPR repeat (Fig. 1a)^22^. Since the CasE ribonuclease of SthCascade is responsible for crRNA maturation and is part of the SthCascade complex (Fig. 1a), each Zm specific spacer sequence was flanked on either side by a CRISPR repeat (Fig. 1b). In this configuration, CasE would excise a mature crRNA from expressed transcripts permitting SthCascade complex formation and target DNA recognition. To enable guide RNA transcription in maize cells, the repeat-Zm spacer-repeat was operably linked to a polymerase III promoter and corresponding terminator from a *Zea mays* U6 gene^30^ residing on chromosome 8 positions 171,225,478–171,226,582 (B73 RefGen_v4 (CSHL)^31^)) of the inbred line B73 (Fig. 1b).

### Type I-E Cascade complex formation and gene activation

**Fig. 2:**
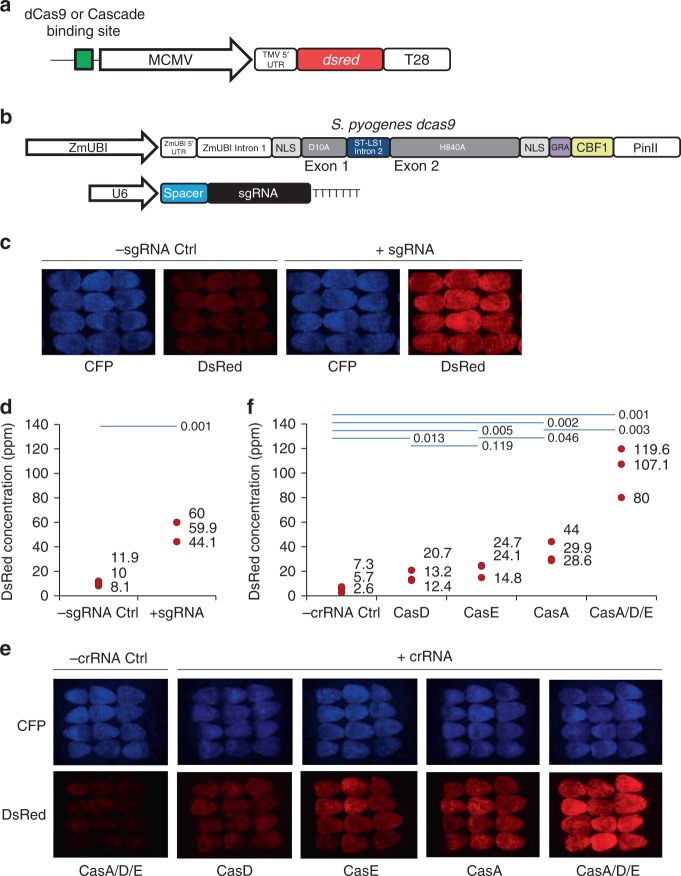
dCas9 and SthCascade *dsred* gene activation in *Zea mays* cells. **a** Expression construct for assaying CRISPR-induced gene activation in *Zea mays*. A single dCas9 or SthCascade binding site upstream of a minimal Cauliflower Mosaic Virus (MCMV) promoter, a Tobacco Mosaic Virus (TMV) 5′ UTR, and a maize codon conditioned gene encoding a variant of the reef coral red fluorescent protein from *Discosoma sp*. (DsRed) followed by a T28 terminator from *Oryza sativa*. **b** *S. pyogenes* dCas9-CBF1 transcriptional activation and sgRNA expression constructs for maize. **c** Confocal immunofluorescence images of dCas9-CBF1 particle gun transformed *Zea mays* immature embryos (IEs). Expression constructs encoding the cyan fluorescent protein (CFP) were co-delivered with other components as a control for transformation efficiency. Experiments assembled without a sgRNA expression cassette served as a negative control (-sgRNA Ctrl). **d** DsRed concentration in dCas9-CBF1 transformed IEs 48 h post transformation. Results from three independent transformations are graphed. Statistical significance between treatments (blue line) was evaluated using a one-sided *t*-test resulting in a *p*-value of 0.001. **e** CFP and DsRed images of SthCascade-CBF1 particle gun transformed IEs. CFP fluorescence served as a control for transformation efficiency. Experiments performed with CasA-CBF1, CasB, CasC, CasD-CBF1, and CasE-CBF1 expression cassettes in the absence of a crRNA expression construct served as the negative control (-crRNA Ctrl). **f** Quantification of DsRed protein resulting from SthCascade transcriptional activation 48 h after transformation in three independent experiments. The *p*-values resulting from a one-sided *t*-test between the indicated treatments (blue lines) are shown

SthCascade complex formation and gene configurations capable of supporting transcriptional activation were confirmed using an immunofluorescent reporter expression cassette (Fig. 2A). It comprised an upstream region where a single SthCascade and crRNA complex may bind, a minimal 35S promoter from the cauliflower mosaic virus (MCMV)^32^, a 5′ untranslated region (UTR) from the tobacco mosaic virus (TMV)^33^, and an open reading frame encoding the DsRed Express fluorescent protein^34^ followed by the T28 terminator from *Oryza sativa*^35^. The reporter construct was validated using *Zea mays* optimized SpCas9 and single guide RNA (sgRNA) expression constructs from Svitashev et al.^29^ except the *cas9* gene was re-engineered to encode a nuclease inactive (dead) Cas9 (dCas9) capable of plant gene transcriptional activation (Fig. 2b). This was achieved by altering codons in the RuvC and HNH nickase domains to encode for alanine residues at positions 10 and 840 of the SpCas9 protein^2,3^ and linking the sequence encoding the plant transcriptional activation domain of CBF1 to the 3′ end of the *cas9* gene (Fig. 2b). Co-delivery of the dCas9-CBF1 cassette, the DsRed reporter construct, and a U6 cassette encoding a SpCas9 sgRNA capable of directing dCas9-CBF1 binding to a region upstream of the MCMV promoter in the reporter construct produced DsRed fluorescence 48 h after microprojectile transformation (Fig. 2c). To confirm visual observations and establish the reproducibility of the assay, the gene product, DsRed protein, resulting from transcriptional activation was quantified using an Octet RED96 system. As shown in Fig. 2d, the amount of DsRed generated among replicates was consistently above the negative control (reactions assembled without the sgRNA) as measured using a one-sided *t*-test with a 0.05 confidence interval.

Next, *Zea mays* optimized SthCascade and crRNA constructs were tested for their ability to support expression, complex formation, and gene activation using the parameters established with dCas9-CBF1. Relative to negative controls (experiments assembled without a crRNA expression cassette), all combinations produced detectable levels of DsRed fluorescence 48 h after transformation (Fig. 2e). Quantification of the amount of DsRed supported visual observations and one-sided *t*-tests further differenitated between treatments showing that CBF1 fusions to CasA produced more DsRed than analogous CBF1 fusions to CasD and CasE (Fig. 2f). Next, we tested if multiple CBF1 domains could be recruited to the DNA target and if gene activation would be impacted. This was accomplished by combining CasA-CBF1, CasD-CBF1, and CasE-CBF1 expression cassettes with the constructs encoding their counterparts, CasB and CasC. After particle gun delivery, the effect was striking resulting in the accumulation of DsRed protein that was greater than that produced individually or by dCas9-CBF1 (Fig. 2c–f). In most replicates, this equated to more DsRed than the sum of that generated for individual CasA-CBF1, CasB-CBF1, and CasE-CBF1 experiments implying a synergistic effect (Fig. 2f).

### Transcriptional activation of an endogenous chromosomal gene

**Fig. 3:**
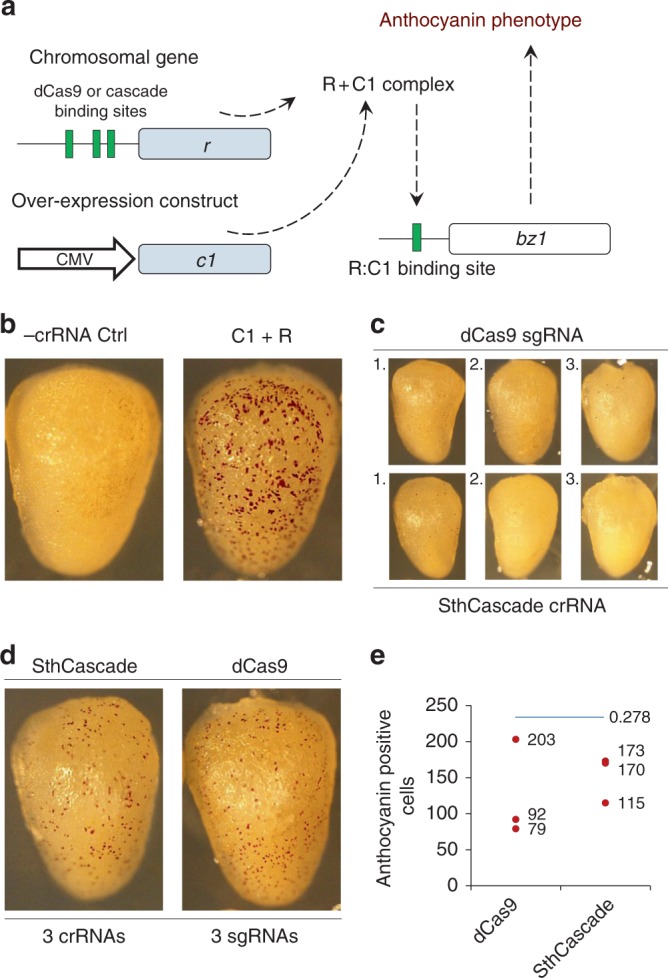
dCas9 and type I-E Cascade chromosomal gene activation in *Zea mays*. **a** Strategy for activating the *Zea mays* anthocyanin pigment pathway in Hi-Type II immature embryos. C1 and R transcription factors complex to activate the *Bronze 1* (Bz1) gene resulting in anthocyanin production. By overexpressing the C1 coding sequence (CDS) using a Cauliflower Mosaic Virus (CMV) enhancer (35S) and promoter (Pro), RNA-guided transcriptional activation of the chromosomal *r* gene results in an anthocyanin pelargonidin (red) cellular phenotype. **b** Negative and positive controls for *r* gene based activation of anthocyanin. The negative control (-crRNA Ctrl) comprised transformations performed with SthCascade expression cassettes (CasA-CBF1, CasB, CasC, CasD-CBF1, CasE-CBF1) and the C1 CDS transgenic over-expression construct in the absence of a crRNA transcriptional cassette. Co-delivery of C1 and R transgenic over-expression cassettes served as a positive control. **c** SthCascade (CasA-CBF1, CasB, CasC, CasD-CBF1, and CasE-CBF1) and dCas9-CBF1 anthocyanin induction 48 h after transformation for each of the corresponding SthCascade cRNAs or dCas9 sgRNAs. **d** SthCascade (CasA-CBF1, CasB, CasC, CasD-CBF1, and CasE-CBF1) and dCas9-CBF1 anthocyanin phenotype when all 3 SthCascade crRNAs or dCas9 sgRNAs were co-delivered. Photos were taken 48 h post-transformation. **e** Quantification of anthocyanin phenotype resulting from SthCascade and dCas9 transcriptional activation. Anthocyanin positive cells were counted on the surface of three independent microprojectile transformations. Treatments were not significantly different based on a one-sided *t*-test (*p* = 0.278)

The ability of SthCascade to access chromosomal DNA and stimulate transcription of an endogenous gene was assessed. Since anthocyanin pigmentation may be used as a visual quantitative marker^36^ for maize transformation, it was selected as a target for endogenous gene activation. In the aleurone cell layer, anthocyanin biogenesis can be activated through the coordinated action of two transcription factors, R and C1, and results in a red pigmented phenotype^37^. Therefore, to induce anthocyanin production, the endogenous *r* gene was targeted for transcriptional upregulation by SthCascade while over-expressing the *c1* gene simultaneously (Fig. 3a). Using dCas9-CBF1, regions receptive to gene activation were established in the R promoter. The most sensitive regions being within 300 bp upstream of the 5′ UTR. Where possible, SthCascade targets that overlapped with the dCas9 sites were then used to compare chromosomal gene activation between the two systems (Supplementary Table 1). In the absence of a sgRNA or crRNA, no change in cellular pigmentation was observed (Fig. 3b). Individually, three different sgRNAs/crRNAs produced little anthocyanin phenotype (Fig. 3c). However, experiments combining all 3 produced the highest levels of anthocyanin production (Fig. 3d). SthCascade and dCas9 yielded near equivalent signal with SthCasacade yielding more consistent chromosomal gene activation (Fig. 3d, e).

## Discussion

Our results establish class 1 type I Cascade complexes as viable tools for RNA guided DNA binding applications. Genes encoding the model type I-E Cascade complex and crRNA from *Streptococcus thermophilus* (Sth) DGCC7710 were conditioned and engineered for expression and nuclear localization in *Zea mays*. By fusing the C-terminal acidic transcriptional activation domain of CBF1 to three Cascade proteins, CasA, CasD, and CasE, we achieved an ~2-fold enhancement in gene activation (as measured by protein accumulation) compared to dCas9. Concatemers and/or multiple combinations of other activators have also been shown in enhance the gene activation potential of Cas9^38^ and could also be applied to SthCascade for an even greater effect. In addition, while we examined the gene activation potential of CasA, CasD, and CasE, greater enhancements may be obtained if the multimeric subunits of Cascade, CasB and CasC, were to be tagged with an activation domain, however, optimal linker sequence and length would likely need to be established. Moreover, since SthCascade experiments require the co-delivery of 6 SthCascade (CasA, CasB, CasC, CasD, CasE, and crRNA) plasmid DNA expression constructs, versus only two cassettes for Cas9, more streamlined delivery strategies such as ribonucleoprotein delivery^39^ or multicistronic expression of the Cascade genes utilizing the self-cleaving 2A peptide^40^ may further enhance the potentcy of SthCascade. Finally, the observed graded response in gene activation (weak for CasD/CasE, moderate for CasA, strong for CasA/CasD/CasE) provides a method to systemically explore different levels of gene activation in a stoichiometric fashion.

Next, we confirmed that SthCascade was able to localize to the nucleus of plant cells. This was accomplished by activating a gene encoding a basic helix-loop-helix transcription factor, R, while simultaneously expressing its Myb-like co-transcription factor C1. SthCascade was able to induce cellular biogenesis of anthocyanin, a red-pigment expressed in the aleurone cell layer found between the seed coat and the endosperm, resulting in a color change of transformed cells. The observed effect over dCas9 was not quite as striking as observed with the immunofluorescent transactivation assay. This could be due in part due to the over-expression of C1 in our assay providing high sensitivity to R gene activation or the ability of SthCascade to access higher ordered eukaryotic DNA. Other type I-E Cascade systems or approaches to increase the DNA binding potential of type I-E Cascade^25^ could provide enhanced target recognition in eukaryotic chromosomal DNA.

Altogether, repurposing type I-E CRISPR-Cascade for use in eukaryotic cells offers great potential to advance genome editing. The overall number of locations available for the acquisition of new domains is potentially 11× compared to Class 2 effectors. For example, in the case of cytosine base editing, both deaminase and uracil glycolase inhibitor (UGI) are needed to provide the utmost potency^9^ and more UGI further enhances base conversion^41,42^. Structural examination of the type I-E Cascade complex from *E. coli* suggests that both N-terminal and C-terminal ends of CasC are exposed along the anterior of the Cascade complex^26^. This indicates that up to 12 UGI domains could easily be recruited to the target site still leaving other Cascade proteins for the recruitment of a deaminase. In addition, DNA nuclease domains could also be associated with Cascade to target chromosomal DNA for cleavage, thereby, enhancing base editing or enabling the introduction of frameshift mutations or modifications by homology directed repair. These may include type II restriction enzyme or homing endonuclease cleavage motifs (e.g., FokI and I-Tev)^43,44^ or simply the type I single stranded exonuclease, Cas3^12^. Moreover, the multi-subunit nature of Cascade affords unique options to control genome editing activity. For example, if a single component is missing, type I-E Cascade DNA target recognition is compromised^16^. This feature could be used to restore activity in desirable spatio-temporal patterns by regulating the expression of the missing component. Also, since the length of guide RNA target recognition is ~10 nts greater than that provided by Class 2 effectors^15,16,22^ and expandable up to 44 nts^25^, DNA target identification by Type I-E Cascade provides greater opportunities for specificity. Finally, comprising more than 50% of all CRISPR-Cas systems identified so far^14^, the diversity afforded by type I systems provides an exceptionally underexplored space for the development of new genome editing tools. Indeed, the simple 5′ PAM recognition reported for type I systems (A or AA for SthCascade)^22^ significantly expands the chromosomal space targetable by CRISPR.

## Methods

### Maize optimized plasmid DNA expression cassettes

*S. thermophilus* DGCC7710 (Sth) *cascade* genes were *Zea mays* codon optimized using *Zea mays* codon tables, optimized for GC content with repetitive sequences and gene destabilizing features such as miniature inverted-repeat transposable elements (MITEs) removed where possible. Overlapping gene fragments were synthesized from GenScript and cloned into expression cassettes containing the *Zea mays* Ubiquitin (UBI) promoter, 5′ untranslated region (UTR), and Intron 1 using the NEBuilder HiFi DNA Assembly kit (New England Biolabs) over BamHI and HpaI sites. Next, sequences encoding a NLS (CasA and CasD expression constructs), the GRA linker, and C-terminal acidic transcriptional activation domain of the cold binding protein 1 (CBF1) from *Arabidopsis thaliana* (AT) were conditioned for *Zea mays* expression and synthesized as described above. The resulting fragments were then seamlessly cloned in-frame at the 3′ end of the *casA*, *casD*, and *casE* genes using a BbsI site incorporated at the end of each gene.

The *S. pyogenes* dCas9 gene activation expression cassette was generated by modifying the *Zea mays* Cas9 expression constructed describer earlier^29^. First, D10A was introduced by exchanging the fragment between the BamHI and SbfI sites of the Cas9 expression cassette with a synthesized fragment (Integrated DNA Technologies (IDT)) encoding the alteration. Next, the sequences encoding H840A, GRA linker, and CBF1 domain were synthesized in two fragments (IDT) and introduced into the D10A cassette by three-piece ligation using AatII, NheI, and PacI sites.

U6 guide RNA expression cassettes were generated by modifying the *Zea mays* U6 guide RNA expression cassette from Svitashev et al.^29^. Both SthCascade crRNA and SpCas9 sgRNA expression constructs were generated by exchanging the sequence between AfeI and EcoRI sites with a fragment(s) encoding the desired guide RNA using a 2-piece or 3-piece ligation scheme. For SthCascade crRNAs, two DNA fragments each containing one CRISPR repeat and part of the sequence encoding the spacer associated with an inverted BbsI site were ligated together with the U6 expression cassette using AfeI, BbsI, and EcoRI sites. SpCas9 sgRNAs were cloned by 2-piece ligation into the U6 expression cassette using AfeI and EcoRI sites.

### Biolistic transformation of maize immature embryos

Particle gun transformation of Hi-Type II 8 to 10-day-old immature embryos (IEs) was carried-out similar to that described in Svitashev et al.^29^. Briefly, DNA expression cassettes were co-precipitated onto 0.6 µM (average size) gold particles utilizing TransIT-2020. Next, DNA coated gold particles were pelleted by centrifugation, washed with absolute ethanol and re-dispersed by sonication. Following sonication, 10 µl of the DNA coated gold particles were loaded onto a macrocarrier and air dried. Next, biolistic transformation was performed using a PDS-1000/He Gun (Bio-Rad) with a 425 lb per square inch rupture disc. Since particle gun transformation can be highly variable, a visual marker DNA expression cassette encoding a cyan fluorescent protein (CFP) was also co-delivered to aid in the selection of evenly transformed IEs and each treatment was performed in triplicate.

### Image analysis of *Zea mays* immature embryos

Two days after biolistic transformation, *Zea mays* immature embryos (IEs) were examined for DsRed and CFP fluorescent signal or anthocyanin pigmentation using a confocal microscope (Nikon AZ100 (Nikon, Japan)). Images of transformed IEs were captured using a Nikon Digital Sight Ds-Fi1 camera (Nikon, Japan) and NIS-Elements BR software (version 4.00.07) (Nikon, Japan).

### Quantification of CRISPR induced protein expression

DsRed expression was quantified using an Octet RED96 System (ForteBio, USA). Total protein was extracted from immature embryos (IEs) two days after transformation and normalized to 1300 µg/ml. Next, a standard curve was generated by examining the binding rate (BR) of purified DsRed protein (Clontech) at known concentrations (500, 100, 25, 6.25 ng/ml) to a DsRed specific biosensor (Anti-Human IgG Fc coated with a DsRed antibody (Clontech)). BRs of the standards were then utilized to generate a standard curve and calculate the concentration of DsRed in the transformed IEs. The BR of each sample including the standards was measured in triplicate and the average used to calculate sample concentration.

### Selection of endogenous *R1* gene activation targets

Three targets were selected in the promoter region of the *r* gene (Zm00001d026147 (GRMZM5G822829)) for both the SthCascade and *Streptococcus pyogenes* dCas9 based on the genomic reference sequence for *Zea mays* B73 (B73 RefGen_v4)^31^. The name and physical position of each target is listed in Supplementary Table 1. Where possible, SthCascade and dCas9 targets were designed in similar locations. Target sequences were confirmed to be identical in Hi-Type II by sequencing.

### Statistics and reproducibility

All experiments were conducted and analyzed with three biological replicates (independent particle gun transformation experiments). To minimize biological variation between experiments, immature embryo isolation was performed in a split-ear fashion (that is all treatments contained an equal number of embryos from the different *Zea mays* ears used). In addition, to ensure unbiased sampling, each replicate was harvested by different technicians. Statistical analyses were conducted using a one-sided *t*-test with a confidence interval of 0.05.

### Reporting summary

Further information on research design is available in the Nature Research Reporting Summary linked to this article.

## Supplementary information

Supplementary Information

Description of additional supplementary items

Supplementary Data 1

Reporting Summary

## Data Availability

All data supporting our study are included in this published article and its supplementary information files. Source data are in Supplementary Data 1. Plasmid DNA expression constructs from our manuscript have been deposited in Addgene (no 132334-132353, see Supplementary Table 2).
