# Supplementary material for: The repurposing of type I-E CRISPR-Cascade for gene activation in plants
Source: Commun Biol. 2019 Oct 18;2:383. doi: 10.1038/s42003-019-0637-6 (PMC6802105; doi:10.1038/s42003-019-0637-6)
Supplement: Supplementary file 1 — Supplementary Information [file 42003_2019_637_MOESM1_ESM.pdf]

Supplementary Tables

Supplementary Table 1. Maize chromosomal DNA targets selected in the promoter region of the *r* gene.

**Name**	**Location (B73 RefGen_v4)**	**PAM (5')**	**Protospacer Target**	**PAM (3')**
crRNA1	Chr 10: 139780885-139780919	AA	GAGGGTCTACTTCCATCACCGTCTTGCTCGGTC	-
crRNA2	Chr 10: 139780833-139780867	AA	CAGCAGTAGTGTTACAGAAGCTAAACTCAACCA	-
crRNA3	Chr 10: 139780942-139780908	AA	TTTATGGACAGAGCTCCAAGTGACCGAGCAAGA	-
sgRNA1	Chr 10: 139780868-139780890	-	GAGCTCCACCAAAGACAAAG	AGG
sgRNA2	Chr 10: 139780672-139780693	-	G*AGTGCTATAATATGAGTGG	TGG
sgRNA3	Chr 10: 139780926-139780905	-	G*TCCAAGTGACCGAGCAAGA	CGG

* Chromosomal target did not contain this G. It was added to the single guide RNA to facilitate robust U6 expression.

Supplementary Table 2. Plasmid DNA expression cassettes used in this study.

Name	Description	Addgene No.
PV225	ZmUBI dCas9-CBF1 expression cassette	132334
PV244	ZmUBI CasB expression cassette	132335
PV245	ZmUBI CasC expression cassette	132336
PV246	ZmUBI CasE expression cassette	132337
PV247	ZmUBI CasA expression cassette	132338
PV248	ZmUBI CasD expression cassette	132339
PV259	ZmUBI CasD-CBF1 expression cassette	132340
PV398	DsRed reporter	132341
PV503	ZmUBI CasE-CBF1 expression cassette	132342
PV504	ZmUBI CasA-CBF1 expression cassette	132343
PV384	ZmU6 Cas9 DsRed targeting sgRNA expression cassette	132344
PV422	ZmU6 SthCascade DsRed targeting crRNA expression cassette	132345
PV521	ZmU6 Cas9 R promoter targeting sgRNA1 expression cassette	132346
PV523	ZmU6 Cas9 R promoter targeting sgRNA2 expression cassette	132347
PV528	ZmU6 Cas9 R promoter targeting sgRNA3 expression cassette	132348
PV564	ZmU6 SthCascade R promoter targeting crRNA1 expression cassette	132349
PV565	ZmU6 SthCascade R promoter targeting crRNA2 expression cassette	132350
PV569	ZmU6 SthCascade R promoter targeting crRNA3 expression cassette	132351

PHP665	C1 over-expression cassette	132352
PHP11004	R over-expression cassette	132353
