# Supplementary material for: The repurposing of type I-E CRISPR-Cascade for gene activation in plants
Source: Commun Biol. 2019 Oct 18;2:383. doi: 10.1038/s42003-019-0637-6 (PMC6802105; doi:10.1038/s42003-019-0637-6)
Supplement: Supplementary file 2 — Description of additional supplementary items [file 42003_2019_637_MOESM2_ESM.docx]

Description of Additional Supplementery Items

Supplementary Data 1. Source Data for figures 2F and 3E
