# Supplementary material for: The repurposing of type I-E CRISPR-Cascade for gene activation in plants
Source: Commun Biol. 2019 Oct 18;2:383. doi: 10.1038/s42003-019-0637-6 (PMC6802105; doi:10.1038/s42003-019-0637-6)
Supplement: Supplementary file 4 — Reporting Summary [file 42003_2019_637_MOESM4_ESM.pdf]

natureresearch

Corresponding author(s): Joshua K. Young

Last updated by author(s): Sep 3, 2019

Reporting Summary

Nature Research wishes to improve the reproducibility of the work that we publish. This form provides structure for consistency and transparency in reporting. For further information on Nature Research policies, see Authors & Referees and the Editorial Policy Checklist.

Statistics

For all statistical analyses, confirm that the following items are present in the figure legend, table legend, main text, or Methods section.

n/a	Confirmed	
☐	☒	The exact sample size (*n*) for each experimental group/condition, given as a discrete number and unit of measurement
☐	☒	A statement on whether measurements were taken from distinct samples or whether the same sample was measured repeatedly
☐	☒	The statistical test(s) used AND whether they are one- or two-sided<br>*Only common tests should be described solely by name; describe more complex techniques in the Methods section.*
☒	☐	A description of all covariates tested
☒	☐	A description of any assumptions or corrections, such as tests of normality and adjustment for multiple comparisons
☐	☒	A full description of the statistical parameters including central tendency (e.g. means) or other basic estimates (e.g. regression coefficient) AND variation (e.g. standard deviation) or associated estimates of uncertainty (e.g. confidence intervals)
☐	☒	For null hypothesis testing, the test statistic (e.g. *F*, *t*, *r*) with confidence intervals, effect sizes, degrees of freedom and *P* value noted<br>*Give P values as exact values whenever suitable.*
☒	☐	For Bayesian analysis, information on the choice of priors and Markov chain Monte Carlo settings
☒	☐	For hierarchical and complex designs, identification of the appropriate level for tests and full reporting of outcomes
☒	☐	Estimates of effect sizes (e.g. Cohen's *d*, Pearson's *r*), indicating how they were calculated

Our web collection on statistics for biologists contains articles on many of the points above.

Software and code

Policy information about availability of computer code

Data collection	Commercially available software associated with the Octet Red96 system was used to determine the concentration of DsRed protein in our samples.
Data analysis	No software was used for data analysis.

For manuscripts utilizing custom algorithms or software that are central to the research but not yet described in published literature, software must be made available to editors/reviewers. We strongly encourage code deposition in a community repository (e.g. GitHub). See the Nature Research guidelines for submitting code & software for further information.

Data

Policy information about availability of data

All manuscripts must include a data availability statement. This statement should provide the following information, where applicable:

- Accession codes, unique identifiers, or web links for publicly available datasets
- A list of figures that have associated raw data
- A description of any restrictions on data availability

All data supporting our study are included in the article (and its supplementary information files). Plasmid DNA expression constructs from our manuscript have been deposited in Addgene , reference numbers 132334-132353 (see Supplementary Table 2). Source data are provided as Supplementary Data.

Field-specific reporting

Please select the one below that is the best fit for your research. If you are not sure, read the appropriate sections before making your selection.

☒ Life sciences ☐ Behavioural & social sciences ☐ Ecological, evolutionary & environmental sciences

For a reference copy of the document with all sections, see nature.com/documents/nr-reporting-summary-flat.pdf

Life sciences study design

All studies must disclose on these points even when the disclosure is negative.

Sample size	No sample size calculation was used for our experiments. Note each transformed immature embryo contains hundreds of independent transformations and a photo or analysis of an immature embryo represents a summation.
Data exclusions	Since plant particle-mediated transformation can be highly variable, uniformly transformed immature embryos were first selected using a fluorescent marker (cyan fluorescent protein). This collection of equally transformed immature embryos were then used in all downstream analyses and comparisons.
Replication	3 independent particle-mediated transformations were setup to account for both technical and biological variation.
Randomization	Immature embryo isolation for particle-mediated transformation was performed in a split-ear fashion (that is all treatments contained an equal numbers of embryos from the different Zea mays ears used in the experiment).
Blinding	Blinding was performed in our study by having independent technicians collect data for each replicate.

Reporting for specific materials, systems and methods

We require information from authors about some types of materials, experimental systems and methods used in many studies. Here, indicate whether each material, system or method listed is relevant to your study. If you are not sure if a list item applies to your research, read the appropriate section before selecting a response.

Materials & experimental systems

n/a	Involved in the study	
☐	☒	Antibodies
☒	☐	Eukaryotic cell lines
☒	☐	Palaeontology
☒	☐	Animals and other organisms
☒	☐	Human research participants
☒	☐	Clinical data

Methods

n/a	Involved in the study	
☒	☐	ChIP-seq
☒	☐	Flow cytometry
☒	☐	MRI-based neuroimaging

Antibodies

Antibodies used	The DsRed Express antibody (Takara Bio formerly Clontech) (catalogue number 632496) was used.
Validation	The antibody was validated against purified DsRed Express protein (Takara Bio formerly Clontech) (catalogue number 632437). This was accomplished by generating a standard curve at known concentrations (500, 100, 25, 6.25 ng/mL) using an Octet RED96 DsRed specific biosensor (Anti-Human IgG Fc loaded with 10μg/ml of the antibody).
